# Some Facts of Interest to Mouth Hygiene Workers

**Published:** 1913-06-15

**Authors:** W. G. Ebersole


### SOME FACTS OF INTEREST TO MOUTH HYGIENE WORKERS.

BY W. G. EBERSOLE, M.D., D.D.S.

In the March issue in a number of dental journals of this Country there appeared an article which we wrote entitled "Some things that should be known about the Mouth Hygiene Movement of this Country." In this article we gave something of a resume of the status of Mouth Hygiene Work in this Country and set forth some of the important features which led to the formation of the National Mouth Hygiene Association which was formed for the purpose of having an organization which would permit the members of all professions and the laity to co-operate in spreading the Mouth Hygiene propaganda.

The work of the Association and its plans and policies were explained and entered into quite extensively. In this connection it was announced that the Secretary-Treasurer had been instructed to conduct a ninety day campaign for the purpose of securing five thousand charter members from the ethical dental profession stating that we desired to do this before beginning an active campaign to secure members from among the laity and other professions. It was also stated that immediately following the ninety day period for securing charter members many of the most prominent and successful workers in philanthropic and uplift organizations would be placed into the field on salary for the purpose of securing membership and funds which would enable the Association to carry out its policies in educating the public to know and understand the value of Mouth Hygiene and to organize the people in the various localities for the purpose of raising funds to establish and maintain suitable dentariums or dental hospitals to care for the worthy poor throughout the country.

At the time this article is being written a little more than half of the ninety day period is past, but the support and co-operation received as the result of our campaign for membership from the dental profession has been such that the Board of Governors felt justified in closing contracts which secured the services of those people whom they believe would increase the working efficiency of the Association to a very marked degree.

First arrangements had been made for the Secretary-Treasurer to devote one-half of his time to the up-building of the organization. One of the most important steps that has been taken in the history of the Association today in the direction of increasing its efficiency has been the engaging of the services of Francis R. Morison of Cleveland as Director of Publicity. Mr. Morison is a publicity expert of some twenty years experience in just those fields that will be of the greatest value to the Association.

Mr. Morison has in his employ twenty people, all of whom are experts in their particular line of publicity.

Mr. Morison's acceptance of publicity directorship places at the disposal of the Association a corps of experts which the Association could not hope to assemble without spending a great deal of time and money in gathering available material.

Committees are being arranged in every state and large city in the Union for the purpose of securing state and city representation in connection with the exhibits. An effort is also being put forth to have the foreign countries who are dealing with Mouth Hygiene represented in connection with this exhibit.

Plans are also under way to secure the active co-operation of the Women's Clubs of America. Through Mrs. S. S. Crockett, Chairman of the Public Health Department of the General Federation of Women's Clubs, over seven thousand Women's Clubs throughout the United States will be invited to co-operation with the Congress on School Hygiene and the result will undoubtedly be of the greatest benefit both to the Congress and to the National Mouth Hygiene Association.

We earnestly solicit the co-operation and support of the dental profession in making a success of this exhibit.

The Fourth International Congress on School Hygiene affords the best opportunity that has ever been offered to the dental profession to place Mouth Hygiene before the public in its true relation to school hygiene in general.

At this meeting will be assembled the largest number of people that have ever gathered in this country for the purpose of considering those questions which deal with School Hygiene. Not only the leading educators and school officials of this country but of the World will be assembled on this occasion. This Congress should be supported by every individual who has interest in the welfare of the public school children of this Country.

